# Protocol for the generation of a human-derived nasal epithelial model and induction of tissue-resident memory-like T cells in a co-culture system

**DOI:** 10.1016/j.xpro.2026.104401

**Published:** 2026-02-26

**Authors:** Lisa A. King, Iris van der Valk, Jasmijn S. Schrumpf, Youvika Singh, Geert H. Groeneveld, Pieter S. Hiemstra, Anne M. van der Does, Simon P. Jochems, Wesley Huisman

**Affiliations:** 1 Leiden University Center for Infectious Diseases, Leiden University Medical Center, Leiden 2333 ZA, the Netherlands; 2 PulmoScience Lab, Department of Pulmonology, Leiden University Medical Center, Leiden 2333 ZA, the Netherlands; 3 Department of Internal Medicine, Leiden University Medical Center, Leiden 2333 ZA, the Netherlands

**Keywords:** cell culture, cell-based assays, flow cytometry, immunology, microscopy, model organisms, single cell

## Abstract

Tissue-resident memory T cells (TRMs) are a distinct subset of T lymphocytes that permanently localize in non-lymphoid tissues and mediate rapid, localized immune responses. Here, we present a protocol to study human TRMs using an *in vitro* model system that induces TRM-like T cells. We describe steps for generating the 3D nasal epithelial model and inducing TRMs via co-culture with autologous immune cells. We then detail procedures to analyze TRMs using flow cytometry, single-cell RNA sequencing, and cytokine release.

## Highlights

• Instructions for generating a 3D nasal epithelial model
• Steps to induce tissue-resident memory-like T cells
• Guidance on analyzing tissue-resident memory-like T cells

## Abstract (editor highlights)

Publisher’s note: Undertaking any experimental protocol requires adherence to local institutional guidelines for laboratory safety and ethics.

## Graphical abstract

**Figure:**
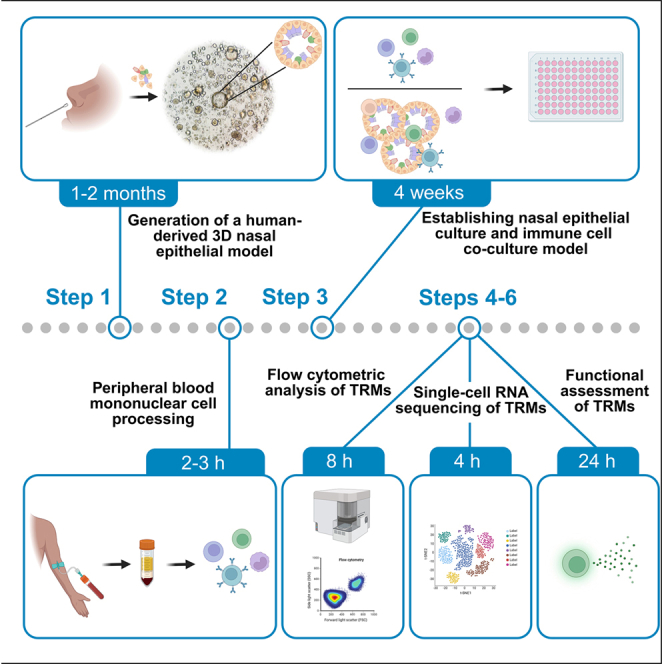

## Before you begin

This protocol provides a detailed description of the generation of a human-derived 3D nasal epithelial model (referred to as 3D NEM) and the induction of TRM-like T cells in an *in vitro* co-culture system. This protocol includes 6 major steps: Generation of a human-derived 3D NEM (steps 1–5); Peripheral blood mononuclear cell processing (step 6); Establishing 3D NEM and immune cell co-culture model (steps 7–8); Flow cytometric analysis of TRMs (steps 9–11); Single-cell RNA sequencing of TRMs (12–15); Functional assessment of *in vitro* established TRM-like cells (steps 16–17).

### Innovation

While 3D cultures of the upper and lower human airways have been published previously, a co-culture model using human-derived nasal epithelium and autologous immune cells that demonstrates TRM induction has not been reported. Since TRM research has primarily focused on *in vivo* data, the ability to mimic human-derived TRM-like cells *in vitro* could accelerate research in diverse contexts, including respiratory infections, vaccines, and immune-related diseases.

### Institutional permissions

Nasal epithelial and peripheral blood mononuclear cell (PBMC) samples were obtained as part of the TINO study, a prospective cohort study investigating the mechanisms and underlying cause of T cell decline with age in the upper-respiratory tract, that was conducted at the Leiden University Medical Center (LUMC, Netherlands). Samples used for the experiments shown in this protocol were derived from healthy young adults (age 18–30). Ethical approval was obtained from the Medical Ethical Committee Leiden-Den Haag-Delft (NL77841.058.21). The trial was registered in the Dutch Trial Registry (NL-OMON24946). All participants provided written informed consent prior to enrollment according to the Declaration of Helsinki.

### Preparation of airway epithelial medium

**Timing: 45 min**

The composition of all media formulations used for this protocol is provided in the materials and equipment section. Here, we provide more details regarding the preparation of the airway epithelial culture medium utilized for the generation and maintenance of a 3D NEM. This medium was originally developed for culturing bronchial epithelial organoid cultures.^1^^,^^2^ Given the similarities between the cell types found in nasal and bronchial epithelia, it was used for our nasal epithelial cell cultures. Successful formation of a 3D epithelial model validated the medium’s applicability, which was successfully adopted in our protocol.

1. Prepare airway epithelial base medium.
  a. Add 5 mL HEPES (1M), 5 mL GlutaMAX (100×) and 1 mL primocin to 500 mL advanced DMEM/F-12.
  b. Once prepared, mix well, and store at 4°C for up to 1 month.
2. Prepare supplements for airway epithelial medium.
  ***Note:*** One Eppendorf tube per aliquoted supplement is enough for 1 batch of 50 mL airway epithelial medium (described under **step** 3).
  a. R-spondin
    i. Dissolve 100 μg R-spondin in 100 μL sterile water.
    ii. Prepare aliquots of 20 μL per Eppendorf tube.
    iii. Store at −20°C for up to 6 months.
  b. FGF-7
    i. Dissolve 2 μg FGF-7 in 20 μL sterile water.
    ii. Prepare aliquots of 10 μL per Eppendorf tube.
    iii. Store at −20°C for up to 6 months.
  c. FGF-10
    i. Dissolve 4 μg FGF-10 in 40 μL sterile water.
    ii. Prepare aliquots of 40 μL per Eppendorf tube.
    iii. Store at −20°C for up to 6 months.
  d. Noggin
    i. Dissolve 4 μg noggin in 40 μL sterile water.
    ii. Prepare aliquots of 40 μL per Eppendorf tube.
    iii. Store at −20°C for up to 6 months.
  e. A83-01
    i. Dissolve 10 mg A83-01 in 4.7 mL sterile dimethyl sulfoxide (DMSO).
    ii. Prepare 10 aliquots of 4 μL and 4 aliquots of ∼1 mL per Eppendorf tube.
    iii. Store at −20°C for up to 6 months.
  f. Y-27632
    i. Dissolve 10 mg Y-27632 in 4 mL sterile DMSO.
    ii. Prepare 10 aliquots of 20 μL and 3 aliquots of ∼1 mL per Eppendorf tube.
    iii. Store at −20°C for up to 6 months.
  g. SB202190
    i. Dissolve 5 mg SB202190 in 3 mL sterile DMSO.
    ii. Prepare 10 aliquots of 4 μL and 3 aliquots of ∼1 mL per Eppendorf tube.
    iii. Store at −20°C for up to 6 months.
  h. B27 supplement
    i. Prepare 12 aliquots of 800 μL per Eppendorf tube.
    ii. Store at −20°C for up to 6 months.
  i. N-acetylcysteine
    i. Prepare fresh when making a new batch of airway epithelial medium.
    ii. Add 20 mg N-acetylcysteine in 1 mL airway epithelial base medium, and incubate for 5–10 min at 37°C to dissolve.
    iii. Filter through a 0.2 μm Whatman filter.
    iv. Add 400 μL per batch airway epithelial medium.
    v. Store at 4°C, check stock for expiry date.
  j. Nicotinamide
    i. Prepare fresh when making a new batch of airway epithelial medium.
    ii. Dissolve 48.8 mg nicotinamide in 1 mL airway epithelial base medium.
    iii. Filter through a 0.2 μm Whatman filter.
    iv. Add 500 μL per batch airway epithelial medium.
    v. Store at 19°C, check stock for expiry date.
3. Prepare airway epithelial medium (one batch).
  ***Note:*** Airway epithelial medium induces both expansion and differentiation of the epithelial cell culture.
  a. Prepare a 50 mL tube with 38 mL airway epithelial base medium.
  b. Thaw 1 aliquot (**step 2)** of R-spondin, FGF-7, FGF-10, noggin, A83-01, Y-27632, SB202190 and B27 aliquots on ice.
  c. Prepare N-acetylcysteine and nicotinamide solutions according to **step 2i** and **2j** and add them to the tube with airway epithelial base medium.
  d. Add, when thawed, 20 μL R-spondin, 10 μL FGF-7, 40 μL FGF-10, 40 μL noggin, 4 μL A83-01, 20 μL Y-27632, 4 μL SB202190, and 800 μL B27 supplement to the tube with airway epithelial base medium.
  e. Mix well and prepare eight aliquots of 5 mL in 15 mL tubes.
    ***Note:*** You need 500 μL of airway epithelial medium per well in a 24-well plate.
  f. Store airway epithelial medium at −20°C for up to 6 months or at 4°C for up to 1 month.

## Key resources table

**Table:** 

REAGENT or RESOURCE	SOURCE	IDENTIFIER
**Antibodies**		

Alexa Fluor 594 rabbit anti-human mucin 5AC (Muc5AC) IgG antibody (final dilution 1:200)	Abcam	ab218363
Alexa Fluor 488 mouse anti-human CC10 IgG1 antibody (final dilution 1:200)	Santa Cruz Biotechnology	sc-365992
Alexa Fluor 647 mouse anti-human acetylated alpha tubulin IgG2b antibody (final dilution 1:200)	Santa Cruz Biotechnology	sc-23950
Alexa Fluor 700 mouse anti-human CD45 IgG1, κ antibody (final dilution 1:800)	BD Pharmingen	560566
Brilliant Violet 711 mouse anti-human CD3 IgG1, κ antibody (final dilution 1:200)	BD Horizon	563725
Brilliant Violet 510 mouse anti-human CD8α IgG1, κ antibody (final dilution 1:200)	BD Horizon	563256
Phycoerythrin-Cyanine 7 mouse anti-human CD4 IgG1, κ antibody (final dilution 1:800)	BD Pharmingen	557852
Fluorescein Isothiocyanate mouse anti-human CD103 IgG1 κ antibody (final dilution 1:20)	BD Pharmingen	550259
Super Bright 780 mouse anti-human CD69 IgG1 κ antibody (final dilution 1:100)	Life Technologies	78-0699-42
Brilliant Violet 650 mouse anti-human CXCR6 IgG2a, κ antibody (final dilution 1:100)	BD OptiBuild	743600
Allophycocyanin mouse anti-human CD62L IgG1 κ antibody (final dilution 1:100)	BioLegend	304810
Allophycocyanine mouse anti-human CD3 IgG2a κ antibody (final dilution 1:20)	BD Pharmingen	555342
Allophycocyanine/Fire 810 mouse anti-human IgG1 κ antibody (final dilution 1:200)	BioLegend	344662
Phycoerythrin mouse anti-human CD8α IgG1 κ antibody (final dilution 1:100)	Life Technologies	12-0088-42
Phycoerythrin-Cyanine 7 mouse anti-human CD69 IgG1 κ antibody (final dilution 1:20)	Life Technologies	25-0699-42
Brilliant Violet 605 mouse anti-human IFNγ IgG1 κ antibody (final dilution 1:200)	BioLegend	502535
Brilliant Ultra Violet 737 mouse anti-human TNFα IgG1 κ antibody (final dilution 1:100)	Life Technologies	367-7349-42

**Biological samples**		

Cryopreserved PBMCs from healthy adults	TINO study (LUMC)	N/A
Nasal epithelial cells from healthy adults	TINO study (LUMC)	N/A

**Chemicals, peptides, and recombinant proteins**		

Phosphate-buffered saline (PBS)	Frensium Kabi	M090001/03
Ethylenediaminetetraacetic acid (EDTA)	Life Technologies	15575–038
Primocin	InvivoGen	ant-pm-2
TrypLE Express Enzyme (1×), phenol red (Tryple E)	Thermo Fisher Scientific	10043382
Cultrex Reduced Growth Factor Basement Membrane Extract, Type 2, Select (BME-2)	Bio-Techne	3536-005-02
Fetal Calf Serum (FCS)	Serana	S-FBS-CO-015
Human serum	Sanquin (contract code: NVT0038)	E8813R00
Recombinant human IL-2 protein	R&D Systems	202-IL-010/CF
Dimethyl Sulfoxide (DMSO)	Sigma-Aldrich	34943-1L
Formalin (formaldehyde solution, 37% in H_2_O)	Sigma-Aldrich	F1635-500mL
Bovine Serum Albumin Fraction V (BSA)	Roche	10735086001
Triton X-100	Sigma-Aldrich	X100-5ML
RPMI 1640 medium, HEPES, no glutamine	Gibco	42401042
Penicillin	Eureco-Pharma	RVG: 125239//120400
Streptomycin	Sigma	S9137-100g
L-glutamin	Sigma	G-8540-100g
Advanced DMEM/F-12	Gibco	12634010
HEPES buffer solution	Gibco	15630056
GlutaMAX	Gibco	35050061
Primocin	InvivoGen	ant-pm-2
Recombinant human KGF (FGF-7)	Tebubio	100–19
Human FGF-10 (FGF-10)	Miltenyi Biotec	130-127-858
Recombinant human noggin	Tebubio	120-10C
Recombinant human R-spondin-1	Tebubio	120–38
A-83-01	Selleckchem	S7692
Y-27632	Sanbio	10005583–10
B-27 supplement	ThermoFisher Scientific	17504044
SB202190 monohydrochloride hydrate	Sigma-Aldrich	S7076-5MG
N-acetylcysteine	Sigma	A9165-5G
Nicotinamide	Sigma	N0636-100G
HBSS, calcium, magnesium	Gibco	24020133
Iscove’s Modified Dulbecco’s Medium (IMDM), with L-Glutamine, without supplements	Capricorn	IMDM-A
4′,6-diamidino-2-phenylindole (DAPI)	Life Technologies	D1306
Live/dead fixable blue dead cell stain kit	Life Technologies	L34962
Phorbol 12-myristate 13-acetate (PMA)	Sigma	P-8139-1mg
Ionomycin	Sigma	I-0634-1mg
Brefeldin A	Sigma	B7651-25mg
Foxp3/Transcription Factor Staining Buffer Set	eBioscience	00-5523-00

**Software and algorithms**		

Fusion Software version 2.6	Oxford Instruments	N/A
Imaris Image Analysis Software version 10.2.0	Oxford Instruments	N/A
Seurat v5		

**Other**		

Rhino-Pro® Curettes	Arlington Scientific	SY-960905
Costar 24-well Clear Not Treated Multiple Well Plates	Corning	3738
Whatman puradisc syringe filters, 0.2 μm pore size	Cytivia	10462200
Vectashield Antifade Mounting Medium	Vector Laboratories	H-1000-10
Superfrost microscope slides	Epredia	AB00008032E01MNZ10
Cover glasses 24×60 mm #1	Epredia	631–1339
Vaseline Original Petroleum Jelly	Vaseline	42182634
Sodium heparin tubes	Greiner	455051
Ficoll amidotrizoate	LUMC Pharmacy	97902861
CompBeads Anti-Mouse Ig, κ/Negative Control Compensation Particles Set	BD	552843
Brilliant Stain Buffer Plus	BD Horizon	566385
Andor Dragonfly 500	Oxford Instruments	N/A
3 laser Cytek Aurora	Cytek	N/A
NovaSeq 6000 PE150bp	Ilumina	N/A
Microseal ‘B' Adhesive Seals	Bio-Rad	MSB1001BEDU
Cell Strainers pore size 40 μm	Corning	CLS352340
Parafilm M Sealing Film	Merck	HS234526A
Nunc cryotubes (1.8 mL)	Thermo Scientific	377267
1.4 mL non coded push cap tubes U-bottom bulk	Micronic	MP32022
Chromium Next GEM single cell fixed RNA sample preparation kit	10× Genomics	1000414
Chromium Fixed RNA kit Human Transcriptome, 4rxns	10× Genomics	1000475

## Materials and equipment

**Table:** Complete RPMI medium

Reagent	Final concentration	Amount
RPMI 1640 medium, HEPES, no glutamine	N/A	499.5 mL
Penicillin (1000000 U)	100 U/mL	250 μL
Streptomycin (20 g)	100 μg/mL	250 μL
**Total**	**N/A**	**500 mL**

***Note:*** Store at 4°C for up to 1 month.

**Table:** Thawing medium

Reagent	Final concentration	Amount
Complete RPMI medium	N/A	45 mL
Heat-inactivated FCS	10%	5 mL
**Total**	**N/A**	**50 mL**

***Note:*** Store at 4°C for up to 1 month.

**Table:** Airway epithelial base medium

Reagent	Final concentration	Amount
Advanced DMEM/F-12	N/A	489 mL
HEPES (1 M)	10 mM	5 mL
GlutaMAX (100×)	1×	5 mL
Primocin (50 mg/mL)	100 μg/mL	1 mL
**Total**	**N/A**	**500 mL**

***Note*:** Store at 4°C for up to 1 month.

**Table:** Airway epithelial medium

Reagent	Final concentration	Amount
Airway epithelial base medium	N/A	38 mL
R-spondin (1000 μg/mL)	25 ng/mL	20 μL
FGF-7 (100 μg/mL)	25 ng/mL	10 μL
FGF-10 (100 ng/μL)	100 ng/mL	40 μL
Noggin (100 μg/mL)	100 ng/mL	40 μL
A83-01 (5 mM)	500 nM	4 μL
Y-27632 (10 mM)	5 μM	20 μL
SB202190 (5 mM)	500 nM	4 μL
B27 supplement (50×)	1×	800 μL
N-acetylcysteine (125 mM)	1.25 mM	400 μL
Nicotinamide (400 mM)	5 mM	500 μL
**Total**	**N/A**	**39.84 μl**

***Note:*** Store at −20°C for up to 6 months or at 4°C for up to 1 month.

**Table:** 0.5% PBS-BSA

Reagent	Final concentration	Amount
PBS	N/A	500 mL
BSA	0.5%	2.5 g
**Total**	**N/A**	**500 mL**

**CRITICAL:** Filter using a 0.2 μm Whatman filter.

***Note:*** Store at 4°C for up to 6 months.

**Table:** Complete HBSS

Reagent	Final concentration	Amount
Hanks’ Balanced Salt Solution	N/A	499.5 mL
Penicillin (1000000 U)	100 U/mL	250 μL
Streptomycin (20 g in 100 mL sterile water)	100 μg/mL	250 μL
**Total**	**N/A**	**500 mL**

***Note:*** Store at 4°C for up to 1 month.

**Table:** T cell base medium

Reagent	Final concentration	Amount
IMDM medium	N/A	494.5 mL
Penicillin (1000000 IU)	100 IU/mL	250 μL
Streptomycin (20 g in 100 mL sterile water)	100 μg/mL	250 μL
L-glutamin	2 mM	5 mL
**Total**	**N/A**	**500 mL**

***Note:*** Store at 4°C for up to 6 months.

**Table:** T cell medium

Reagent	Final concentration	Amount
T cell base medium	N/A	42.5 mL
IL-2 (91000 IU)	50 IU/mL	2.5 μL
Heat-inactivated FCS	5%	2.5 mL
Heat-inactivated human serum	5%	2.5 mL
**Total**	**N/A**	**50 mL**

**CRITICAL:** Prepare fresh.

***Note:*** Store at 4°C for up to 1 month.

**Table:** FACS buffer

Reagent	Final concentration	Amount
PBS	N/A	498 mL
EDTA (0.5 M)	2 mM	2 mL
BSA	0.5%	2.6 G
**Total**	**N/A**	500 mL

**CRITICAL:** Filter using a 0.2 μm Whatman filter.

***Note:*** Store at 4°C for up to 6 months.

## Step-by-step method details

### Generation of a human-derived 3D nasal epithelial model

**Timing: 1–2 months (highly dependent on donor)**

**Timing: 2 h (for step 1)**

**Timing: 5–15 min (dependent on the amount of wells) (for step 2)**

**Timing: 2 h (dependent on the amount of wells) (for step 3)**

**Timing: 1.5 h (dependent on the amount of cells) (for step 4)**

**Timing: 2 days (for step 5)**

This section outlines a step-by-step protocol for the initiation of nasal curettage-derived cells into a human-derived 3D NEM. It includes detailed procedures for replacing medium, passaging, cryopreservation and thawing, as well as the characterization of differentiated nasal epithelial cultures.

**Figure 1:**
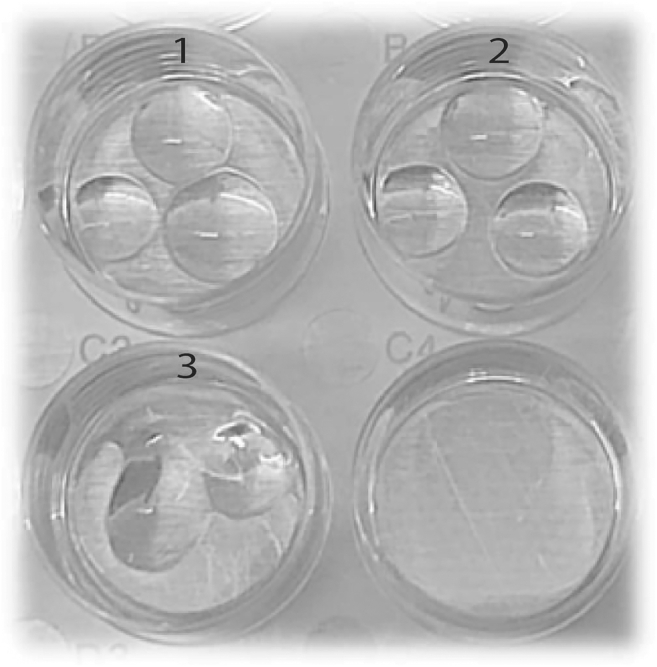
BME-2 droplets in 24-well plate Three BME-2 droplets containing nasal epithelial cells were dispensed into three wells of a 24-well plate. Wells 1 and 2 are examples of well-separated droplets, and well 3 is an example showing merged droplets.

**Figure 2:**
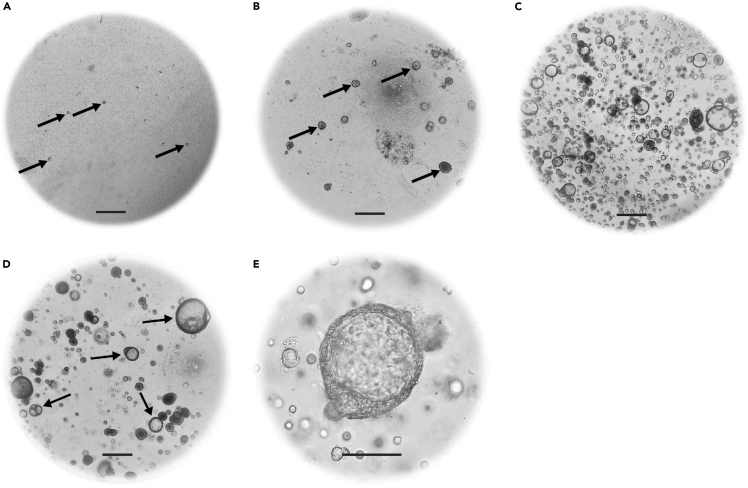
Representative images of 3D NEM (A and B) Representative brightfield images of a 3D NEM cultured for (A) 2 days or (B) 7 days post initiation culture. (5× magnification). Black arrows highlight 3D NEM (still undifferentiated). (C) Brightfield image of a 3D NEM cultured for 2 weeks and ready to be passaged (5× magnification). (D and E) Brightfield images of a representative 3D NEM passaged four times and differentiated for 2 weeks. Differentiated 3D structures are indicated by black arrows (5× magnification). (E) Higher magnification (10×) image of a differentiated 3D nasal epithelial cell structure. The black scale bars indicate 30 μM.

**Figure 3:**
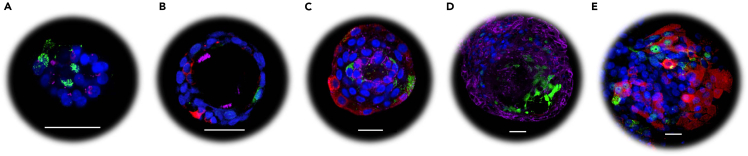
Differentiated 3D NEM (A–E) Representative confocal microscopy images of fixed and permeabilized 3D nasal epithelial structures (n=2), following a (A) 1, (B) 2, (C) 3 or (D, E) 4-week differentiation period (without passaging). The 3D nasal epithelial structures were stained with DAPI (blue, cell nuclei) and Muc5AC (red, goblet cells), acetylated alpha tubulin (purple, cilia/ciliated cells) and CC10 (green, club cells) fluorescently labelled antibodies. Z-stack imaging was used to capture the entire 3D structure. The 40× oil objective was used and the white scale bars indicate 30 μM.

1. Initiation of a human 3D nasal epithelial model
  ***Note:*** Successful initiation of nasal epithelial cells into a 3D NEM varies among donors and is dependent on the amount and quality of epithelial cells in the starting material.
  a. Pre-warm a 24-well flat bottom plate in an incubator (37°C, 5% CO_2_).
  b. Pre-warm airway epithelial medium at 37°C.
    ***Note:*** Calculate the amount of medium needed and pre-warm that exact amount. You need 500 μL of airway epithelial medium per well in a 24-well plate.
  c. Sample both nostrils using 1 nasal curette per side.
    i. Scrape with a nasal curette 2–3 times gently over the nasal mucosa of the inferior turbinate.^3^
      ***Note:*** Nasal cells collected with other minimally-invasive methods, such as nasopharyngeal flocked swabs,^4^ can also be used as starting material for a 3D NEM.
  d. Collect nasal curettes in pre-cooled 15 mL canonical tubes containing 8 mL sterile PBS supplemented with 0.5% heat-inactivated fetal calf serum (FCS) and 5 mM ethylenediaminetetraacetic acid (EDTA).
    ***Note:*** Primocin can be added to prevent microbial growth.
  e. Quickly move the nasal curettes around in the supplemented PBS to release cells.
  f. Pipet 500 μL supplemented PBS from the tube onto the top of the nasal curettes to release remaining cells.
  g. Repeat **step 1f** four times.
  h. Centrifuge the 15 mL canonical tubes at 4°C, 230 × *g* for 7 min.
  i. Pipet off supernatant until approximately 40 μL is left.
  j. Resuspend cell pellets and combine cells of right and left nostrils (80 μL cell suspension in total).
  k. Resuspend cells in 500 μL TrypLE Express Enzyme (Tryple E).
    ***Note:*** Tryple E is used to obtain a single cell suspension.
  l. Incubate cells at 37°C for 7 min.
  m. Dissociate again by repeating **steps 1k-l**, resuspend the cell suspension by pipetting at least 5 times up and down when adding 500 μL Tryple E.
    ***Note:*** A better single cell suspension is obtained by adding 2 times 500 μL over adding 1 mL for 14 min.
  n. Add 2 mL complete RPMI medium supplemented with 10% heat-inactivated FCS to inactivate the Tryple E and resuspend well.
  o. Centrifuge the 15 mL canonical tube at 19°C, 230 × *g* for 7 min.
    i. During centrifugation, place an aliquot containing Cultrex Reduced Growth Factor Basement Membrane Extract, Type 2, Select (BME-2) on ice until needed.
  p. Carefully aspirate the supernatant without disrupting the cell pellet.
    ***Note:*** At this stage, the cells form a loose pellet. If the pellet comes loose at **step 1p**: pipet back the detached cells and repeat **steps 1o and p**.
  q. Centrifuge the 15 mL canonical tube at 19°C, 230 × *g* for 1 min.
  r. Remove the remaining supernatant completely with a 200 μL pipet to obtain a ‘’dry’’ pellet.
    **CRITICAL:** It is crucial to ensure that the cell pellet is completely free of residual supernatant prior to the addition of BME-2 to avoid compromising its structural properties, which are essential for optimal 3D NEM.
  s. Resuspend the cell pellet in cold 90 μL BME-2, pipet very slowly up and down to avoid creating air-bubbles.
    ***Note:*** To avoid bubbles, it is recommended to use two sets of 100 μL pipettes, one set at 90 μL to add the BME-2, the other set at 50 μL used to resuspend. Alternatively Eppendorf tubes can also be used to improve accessibility.
  t. Store the cells on ice and wait approximately 5 min or until the tube feels cold.
    ***Note:*** BME-2 solidifies at temperatures above 4°C. Therefore, maintaining the BME-2- diluted cells on ice facilitates easier pipetting.
  u. Collect the pre-warmed flat-bottom 24-well plate from the incubator (37°C, 5% CO2).
  v. Pipet the BME-2 resuspended cells as three individual droplets (30 μL each) in one 24-well, without creating air-bubbles, as illustrated in Figure 1 (see troubleshooting problem 1 for more information).
  w. Carefully place the 24-well plate in the incubator (37°C, 5% CO_2_) to allow the droplets to solidify.
  x. Wait approximately 5 minutes, and add 500 μL pre-warmed airway epithelial medium slowly along the side of the 24-well.
  y. Incubate the 24-well plate in an incubator (37°C, 5% CO_2_).
    i. Check the cells daily using brightfield microscopy.
      ***Note:*** Formation of 3D structures typically start to appear within 1–2 days and increase in size over time (Figures 2A–2E). The culture is deemed unsuccessful if 3D structures don’t appear within 7 days.
2. Replacing airway epithelial medium.
  ***Note:*** Replace airway epithelial medium every 3–4 days.
  a. Pre-warm airway epithelial medium at 37°C.
    ***Note:*** Calculate the amount of medium needed and pre-warm that exact amount.
  b. Collect the 24-well plate from the incubator and carefully remove the medium from the well(s) containing 3D NEM, ensuring not to disrupt the BME-2 droplets.
  c. Add 500 μL warm airway epithelial medium slowly along the side of a 24-well.
  d. Incubate the 24-well plate in an incubator (37°C, 5% CO_2_).
3. Cryopreserving and passaging 3D NE.
  ***Note:*** 3D NEM will start to differentiate after two weeks in culture. Passaging and/or cryopreserving should therefore be done before this time-point during the expansion phase. Passage the cell culture at least once, in order to get rid of debris/ dead cells, before cryopreserving the epithelial cells. Keep cells in culture for two weeks before passaging them, unless the medium turns yellow (too many cells) before medium refreshment and/or if the cell culture is confluent (organoids touching each other and little/ no open space left, Figure 2C).
  **CRITICAL:** BME-2 is extremely viscous, which may result in the loss of cells during handling. To minimize this, it is recommended to pre-coat plastic tubes with airway epithelial base medium. In case of pipet tips, pipette first the airway epithelial base medium up and down to coat the inside of the tip before pipetting the 3D NEM.
  a. Cool a bottle of sterile PBS to 4°C.
  b. Pre-warm a new 24-well flat bottom plate in an incubator (37°C, 5% CO_2_).
  c. Pre-warm airway epithelial medium at 37°C.
    ***Note:*** Calculate the amount of medium needed and pre-warm that exact amount.
  d. Collect the 24-well plate containing 3D NEM from the incubator and carefully remove the airway epithelial medium from the wells, ensuring not to disrupt the BME-2 droplets.
  e. Add 1 mL cold PBS per 24-well, resuspend by pipetting 5 times up and down until the BME-2 has been dissolved and gently scrape the bottom of the well with the pipette tip to remove BME-2 remnants.
  f. Transfer the 3D NEM to a 15 mL canonical tube and wash the wells with an additional 1 mL of cold PBS, and transfer to the corresponding tube.
  g. Add cold airway epithelial base medium until reaching 15 mL and centrifuge at 4°C, 230 × *g* for 7 min.
    **CRITICAL:** Check after centrifuging if the 3D nasal epithelial structures are pelleted and that none are in the BME-2 remnant above the pellet. If a clear pellet is visible, pipet off the supernatant including BME-2 layer. If 3D nasal epithelial structures are in the BME-2 remnant, centrifuge again and remove supernatant and BME-2 layer.
  h. Carefully aspirate the supernatant without disrupting the cell pellet.
  i. Dissociate the 3D nasal epithelial structures according to **steps 1k-p**.
  j. Resuspend the epithelial single cell suspension in 2 mL airway epithelial base medium.
  k. Count the epithelial single cell suspension.
    i. Mix 20 μL cell suspension with 20 μL trypan blue and transfer 10 μL into a Bürker Türk counting chamber.
      ***Note:*** Decide if you want to cryopreserve (continue with **step** l) and/or passage epithelial cells (continue with **step** m). For each 24 wells well with 3 droplets an expected yield between 0.5 × 10^6^ and 2 × 10^6^ can be expected, depending on donor.
  l. Cryopreserve epithelial cells if sufficient cell numbers are available to freeze at least 100,000 cells per Nunc cryotube. It is recommended to have low passage numbers of epithelial cells available in the liquid nitrogen.
    i. Resuspend the epithelial single cell suspension in 100,000 cells per 500 μL cold complete RPMI medium supplemented with 20% FCS.
    ii. Slowly add, dropwise, 500 μL cold complete RPMI medium supplemented with 20% FSC and 20% DMSO per 100,000 cells.
      ***Note:*** DMSO is toxic for cells; proceed with the subsequent steps as quickly as possible to minimize poor cell viability.
    iii. Transfer 1 mL epithelial single cell suspension per Nunc cryotube.
    iv. Place Nunc cryotubes in a pre-chilled (4°C) Mr. Frosty freezer container and transfer to a −70 or −80°C freezer.
    v. After a minimum of 24hrs, transfer Nunc cryotubes to the liquid nitrogen for long term storage.
  m. To passage epithelial cells, centrifuge cells at 19°C, 230 × *g* for 7 min.
    i. Collect an aliquot containing BME-2 and keep on ice until needed.
    ii. Obtain a ‘’dry’’ cell pellet according to **steps 1p-r.**
    iii. Resuspend cell pellet in cold BME-2 (50,000 epithelial cells/30 μL BME-2), pipet very slowly to avoid creating air-bubbles.
    iv. Initiate 3D NEM by repeating **steps 1t-y**.
      ***Note:*** We have successfully performed assays with passages up to 10, we recommend to not exceed this passage number or to test effects on assays performed when exceeding this number
4. Thawing and culturing cryopreserved nasal epithelial cells.
  a. Pre-warm thawing medium at 37°C.
  b. Collect an aliquot containing BME-2 and keep on ice until needed.
    ***Note:*** Thaw no more than two vials per person at a time to ensure proper handling.
  c. Retrieve Nunc cryotubes from liquid nitrogen storage, transfer them on dry ice or in liquid nitrogen, and thaw in a 37°C water bath until a small ice clump (pea-sized) remains.
  d. Transfer the cells from the Nunc cryotubes into a 15 mL canonical tube.
  e. While gently moving the tube, slowly add 13 mL of pre-warmed thawing medium dropwise.
  f. Rinse the Nunc cryotubes with 1 mL of pre-warmed thawing medium and transfer this to the same 15 mL tube.
  g. Centrifuge the 15 mL canonical tube at 19°C, 230 × *g* for 7 min.
  h. Carefully aspirate as much of the supernatant as possible without disrupting the cell pellet.
  i. Resuspend the cell pellet with 15 mL pre-warmed thawing medium.
  j. Initiate 3D NEM by repeating **steps 1o-y.**
    i. Use 30 μl BME-2 per Nunc cryotube (if 100,000 cells are stored per cryotube).
5. Characterization of differentiated 3D NEM using confocal microscopy
  ***Note:*** Characterize differentiation state of 3D NEM when cultured for 2–4 weeks without passaging them in between.
  **CRITICAL:** BME-2 is extremely viscous, which may result in the loss of cells during handling. To minimize this, it is recommended to pre-coat plastic tubes with airway epithelial base medium. In case of pipet tips, pipette first the airway epithelial base medium up and down to coat the inside of the tip before pipetting the 3D NEM.
  a. Visualize the 3D NEM in 24-well plates using brightfield microscopy (e.g., a standard inverted microscope) and assess if the majority of structures display a lumen (indicative of differentiation^5^^,^^6^), as shown in Figures 2D and 2E.
    ***Note:*** Extend the duration of the culture if the majority of structures do not display a lumen 2 weeks post-culture initiation.
  b. Carefully remove the airway epithelial medium from the wells, ensuring not to disrupt the BME-2 droplets.
  c. Add 750 μL cold PBS per 24-well, resuspend by pipetting 5 times up and down until the BME-2 has been dissolved and gently scrape the bottom of the well to remove BME-2 remnants.
  d. Transfer the cells to a 1.5 mL Eppendorf tube and wash the well with an additional 500 μL of cold PBS, and transfer to the same Eppendorf tube.
  e. Centrifuge at 19°C, 500 × *g* for 5 min.
  f. Carefully remove the supernatant and fixate cells for 1 h at 19°C with 1.5 mL 4% formalin in PBS.
  g. Centrifuge at 19°C, 500 × *g* for 5 min.
  h. Carefully remove most of the formalin.
    **Pause point:** Store the fixated cells in 500 μL PBS at 4°C for maximum 7 days. In case longer storage is preferred then fixated cells may be kept in 70% ethanol at 4°C for up to 1 month.^7^
  i. Resuspend the fixated cells in 1 mL PBS supplemented with 0.5% PBS-BSA and centrifuge at 19°C, 500 × *g* for 5 min.
  j. Carefully remove the supernatant and permeabilize the cells with 200 μL 0.5% Triton-X100 diluted in 0.5% PBS-BSA.
  k. Place Eppendorf tubes inside a 50 mL tube and incubate for 30 min at 19°C on the roller bank.
  l. Add 1 mL 0.5% PBS-BSA and centrifuge at 19°C, 500 × *g* for 5 min.
  m. Carefully remove the supernatant and block with 1.0 mL 0.5% PBS-BSA.
  n. Place Eppendorf tubes inside a 50 mL tube and incubate for 30 min-1 h at 19°C on the roller bank.
  o. Centrifuge at 19°C, 500 × *g* for 5 min.
  p. Carefully remove the supernatant and resuspend cells in 200 μL microscopy staining mix (Table 1) per sample.
    ***Note:*** If preferred, other fluorochromes can be used.
  q. Place the Eppendorf tubes into a 50 mL tube, wrap the outside of the 50 mL tube in aluminum foil and incubate for 16 h at 4°C on a roller bank.
  r. Wash stained cells by adding 1 mL 0.5% PBS-BSA to the tube.
  s. Centrifuge at 19°C, 500 × *g* for 5 min.
  t. Carefully remove the supernatant and wash again with 1.5 mL 0.5% PBS-BSA.
  u. Centrifuge at 19°C, 500 × *g* for 5 min.
  v. Carefully remove the supernatant and resuspend cells in 200 μL 0.5% PBS-BSA supplemented with 1000× diluted DAPI per sample.
  w. Place Eppendorf tubes inside a 50 mL tube, wrap the tube in aluminum foil and incubate 1.5–2 h at 19°C on a roller bank.
  x. Add 1 mL 0.5% PBS-BSA and centrifuge at 19°C, 500 × *g* for 5 min.
  y. Carefully remove the supernatant and wash again with 1.5 mL 0.5% PBS-BSA and centrifuge at 19°C, 500 × *g* for 5 min.
  z. Carefully remove the supernatant, resuspend cells in a single droplet of Vectashield Antifade Mounting Medium, and transfer the suspension onto a glass microscope slide.
  aa. Apply a small amount of Vaseline to the corners of a coverslip and place it over the cells on the glass microscope slide.
  bb. Image the 3D nasal epithelial structures, it is preferable to use a confocal microscope system that can acquire Z-stacks to be able to analyze the whole 3D structure (e.g., the Confocal Andor Dragonfly 500 combined with Fusion Software).
    i. Visualize the 3D structures with a 10× objective/lens and subsequently use a 40× oil immersion objective/lens to get more detailed images.
    ii. Activate the correct channels (Table 2).
    iii. Optimize the exposure time and laser power per channel.
    iv. Activate the Z-stack mode, define the upper (top of the structure) and lower (bottom of the structure) limits (depends on the size of the structure), determine the step size (we recommend 20 steps) and capture the images. Repeat for other structures.
    v. Finalize and export the images, we used Imaris Image Analysis Software (version 10.2.0).
      ***Note:*** As illustrated in Figure 3, differentiated nasal epithelial structures contain increased amounts of club cells (CC10), goblet cells (Muc5AC) and ciliated cells/ cilia (acetylated alpha tubulin) over the course of 4 weeks. Well differentiated structures contain a lumen with inward-facing ciliated cells/cilia, which is visible after 2 weeks of differentiation, and contain more ciliated cells/cilia over time (Figures 3B–3D).

**Table 1:** Preparation of microscopy staining mix

Reagent	Volume (μL/reagent)
Alexa Fluor 488 mouse anti-human CC10 IgG1 antibody (1:200)	1
Alexa Fluor 594 rabbit anti-human mucin 5AC IgG antibody (1:200)	1
Alexa Fluor 647 mouse anti-human acetylated alpha tubulin IgG2b antibody (1:200)	1
0.5% PBS-BSA in PBS	197
**Total**	**200**

**Table 2:** Details settings confocal microscope

Marker + fluorochrome	(Excitation/laser) + channel	Filter
CC10 (Alexa Fluor 488)	(488)_GFP_CF40_Zyla	525/50
Mucin 5AC (Alexa Fluor 594)	(561)_RFP_CF40_Zyla	620/60
Acetylated alpha tubulin (Alexa Fluor 647)	(637)_Cy5_CF40_Zyla	700/75
DAPI	(405)_DAPI_CF40_Zyla	450/50

### Peripheral blood mononuclear cell processing

**Timing: 2–3 h**

This section provides a step-by-step protocol of the isolation of PBMCs.

**Figure 4:**
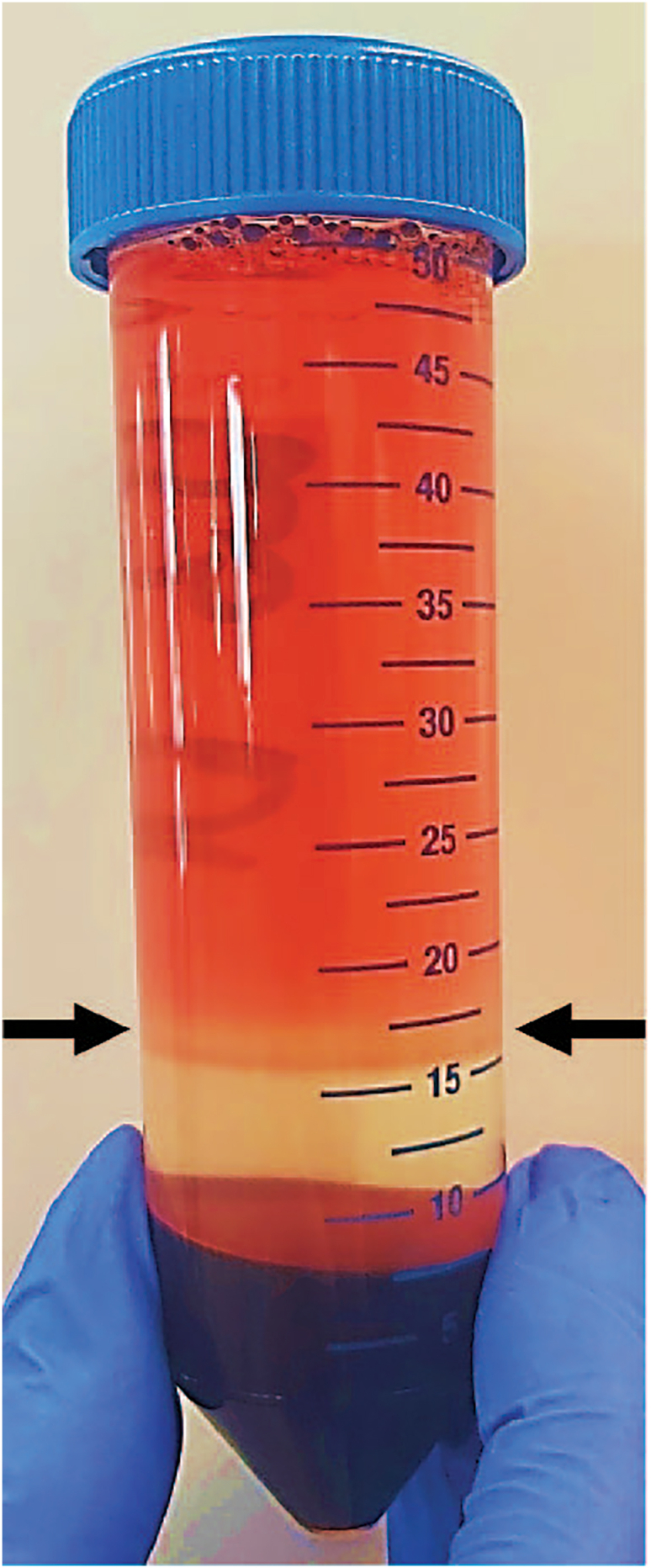
White interphase PBMC layer after Ficoll seperation Representative picture of white interphase PBMC layer highlighted by the 2 black arrows, after centrifugation.

6. Isolation of PBMCs
  ***Note:*** Ensure that **steps 6a-k** are performed at 19°C.
  a. Transfer heparinized blood from four 10 mL sodium heparin tubes to two canonical 50 mL tubes.
  b. Dilute the blood 1:1 with complete HBSS.
  c. Carefully layer 10 mL Ficoll beneath the diluted blood.
    i. Using a Pipetboy and a 10 mL serological pipette, aspirate 13 mL of Ficoll (the last 3 mL of Ficoll stays in the pipet) and gently place the pipette tip on the bottom of the tube.
    ii. Take off the pipet from the Pipetboy and let the ficoll dispense under the blood.
    iii. Once 10 mL of Ficoll has been fully dispensed, place a finger over the top of the pipette and carefully remove the pipette from the tube.
  d. Centrifuge at 19°C, 400 × *g* for 25 min and set brake at 0.
  e. Remove the top layer until ∼1 mL above the white interphase PBMC layer (Figure 4).
  f. Carefully transfer the white interphase PBMC layer (on top of the Ficoll, Figure 4) of two 50 mL tubes to a new 50 mL tube.
  g. Add complete HBSS until 50 mL.
  h. Centrifuge at 19°C, 500 × *g* for 10 min.
  i. Remove the supernatant and resuspend the PBMC pellet in 50 mL complete HBSS.
  j. Centrifuge at 19°C, 450 × *g* for 5 min.
  k. Count the PBMCs (see **step 3k** for details regarding counting).
  l. Freeze PBMCs in 5–10 × 10^6^ cells per Nunc cryotube (see **step 3l** regarding cryopreservation).

### Establishing the 3D NEM and immune cell co-culture model

**Timing: 4 weeks**

This section provides a step-by-step protocol of the initiation and maintenance of the *in vitro* co-culture model combining the 3D NEM with autologous immune cells. Use 3D NEM that have been differentiated for at least two weeks, and confirm differentiation status with the microscopy staining described in section 5.

7. Harvesting 3D NEM
  a. Prepare a tube of T cell medium (see materials and equipment) and pre-warm it to 37°C.
  b. Collect 3D NEM from BME-2 by following **steps 3d-h.**
  c. Resuspend pellet in 1 mL pre-warmed T cell medium.
  d. Transfer 50 μL to a new tube and store the rest in the incubator (37°C, 5% CO_2_).
    ***Note:*** A part of the 3D NEM (50 μL) is made into a single cell suspension and epithelial cells are counted to determine the total amount of epithelial cells.
  e. Add 500 μL Tryple E to the tube with 50 μL of the suspension.
  f. Repeat steps **1k-p.**
    ***Note:*** BME-2 is not needed.
  g. Resuspend the cell suspension in 1 mL warm T cell medium.
  h. Count the cells (**step 3k**) and calculate how many epithelial cells there are in total.
    i. Example calculation:
      Counts: 100,000 epithelial cells counted in 1 mL. This aliquot was 1/20^th^ of the original cell suspension. The cell suspension (**step 7c**) contains therefore 100,000 · 19 = 1.9 · 10^6^ epithelial cells.
  i. Resuspend at 200.000 cells/mL in warm T cell medium.
  j. Pipet 100 μL cell suspension per 96-well (flat bottom).
    ***Note:*** 100 μL cell suspension contains 20.000 epithelial cells per condition.
    i. Pipet in triplicate for each donor.
  k. Incubate the 96-well plate(s) in the incubator (37°C, 5% CO_2_).
    ***Note:*** The cells are kept warm while the PBMCs are being prepared (**step 8**).
8. Initiation and maintenance of the 3D NEM and immune cell co-culture
  a. Thaw cryopreserved autologous PBMCs by following **steps 4a-h.**
  b. Centrifuge the 15 mL canonical tube at 19°C, 230 × *g* for 7 min.
  c. Carefully aspirate as much of the supernatant as possible without disrupting the cell pellet.
  d. Resuspend PBMCs at 1 · 10^6^ cells/mL warm T cell medium.
  e. Collect the 96-well plate(s) from the incubator and pipet 100 μL PBMC suspension per 96-well to obtain a PBMC control condition and a PBMC + 3D NEM condition.
    i. 100 μL PBMC suspension contains 100,000 cells per condition.
    ii. Pipet 100,000 PBMCs to each well containing epithelial cells and also include a PBMC only control (in triplicate).
  f. Add warm T cell medium to achieve a final volume of 200 μL per well in the control conditions.
  g. Add 200 μL of PBS into the outer wells of the 96-well plate(s) to reduce evaporation in culture conditions during incubation, which is known as the ‘edge effect’.^8^
  h. Incubate the 96-well plate(s) in the incubator (37°C, 5% CO_2_).
  i. Change T cell medium every 2–3 days.
    i. Carefully remove 50 μL T cell medium per well.
    ii. Add 50 μL warm T cell medium per well; make sure to not disturb the cells by pipetting the medium near the top along the walls into the wells.
  j. Perform flow cytometric analysis of the TRM phenotype of T cells at baseline and subsequently on days 7, 14, 21, and 28 post-start culture, as described in **section** flow cytometric analysis of TRMs**.**

***Note:*** Optional, perform single cell RNA sequencing (scRNAseq) and gene set enrichment analysis (GSEA) of the TRM phenotype of T cells at baseline and day 14 or day 21 post-start culture.

### Flow cytometric analysis of TRMs

**Timing: Variable depending on the number of experimental conditions and/or donor and the staining protocol; maximum duration is 8 h, including acquisition on the Cytek Aurora**

This section outlines a detailed protocol for the weekly flow cytometric analysis of PBMCs at baseline (pre-culture) and the longitudinal assessment of TRMs in the *in vitro* established cultures.

We also describe the preparation of reference controls required for spectral unmixing. These include an unstained control and a series of single-stained controls corresponding to each fluorochrome used in the panel. Whenever feasible, these controls should be prepared using the same cell type(s) present in the experimental samples to ensure spectral accuracy. However, in cases where cell populations are rare, target antigen expression is low or absent, or sample availability is limited, compensation beads (e.g., BD CompBeads) can be used as a practical alternative for generating reference controls. Reference controls can be made once and then re-used for the duration of the experiment.

9. Staining PBMCs at start culture (baseline measurement)
  a. Transfer 100,000 PBMCs per donor per well in a 96-well V-bottom plate.
  b. Top up with PBS until 200 μL per 96-well.
  c. Centrifuge at 19°C, 450 × *g* for 5 min.
  d. Remove supernatant and add 200 μL PBS per 96-well.
    ***Note:*** Remove supernatant by flipping the 96-well plate over a sink with one sharp hand movement, and directly pat the 96-well plate dry on tissue paper.
  e. Centrifuge at 19°C, 450 × *g* for 5 min.
  f. Remove supernatant and add 100 μL of live-dead staining mix (Table 3) per sample.
    ***Note:*** If preferred, another viability dye can be used.
  g. Cover the plate with aluminum foil and incubate at 19°C for 15 min.
  h. Add 100 μL FACS buffer per 96-well.
  i. Centrifuge at 19°C, 450 × *g* for 5 min.
  j. Remove supernatant and add 200 μL FACS buffer per 96-well.
  k. Centrifuge at 19°C, 450 × *g* for 5 min.
  l. Remove supernatant and add 100 μL of membrane staining mix (Table 4) per sample.
    ***Note:*** If preferred, other fluorochromes can be used.
  m. Wash away excess staining by repeating **steps 9h-k.**
  n. Remove supernatant, resuspend cell pellets in 150 μL FACS buffer and transfer to 1.4 mL U-bottom tubes.
  o. Measure the cells with a Cytek Aurora.
    i. Transfer the 1.4 mL U-bottom tubes to 5 mL FACS tubes when acquiring.
10. Harvesting, staining and measuring TRMs, 7, 14, 21 and 28 days post-start culture
  a. Collect the plate(s) from the incubator and centrifuge at 19°C, 450 × *g* for 5 min.
    i. Optional: Collect ∼150 μL culture supernatant in 96-well round bottom plate(s), seal with a plate seal, add the lid on top and store at −20°C. If not needed, discard supernatant.
  b. Resuspend the cells with 150 μL PBS and transfer them through a 40 μm strainer in a new conical (V)-bottom plate.
    ***Note:*** 3D epithelial cell structures are removed with the 40 μm strainer from PBMCs as they will clog the Cytek Aurora during acquisition.
  c. Wash strainers with 50 μL PBS (endvolume per 96-well is 200 μL) to get remaining immune cells and discard strainer (containing 3D epithelial cell structures).μLμL
  d. Centrifuge plate(s) at 19°C, 450 × *g* for 5 min.
  e. Remove supernatant, and add 200 μL PBS per 96-well.
  f. Centrifuge at 19°C, 450 × *g* for 5 min.
  g. Stain the cells according to **steps 9f-o.**
11. Preparation reference controls for Cytek Aurora
  a. Transfer 100,000 PBMCs (directly after thawing, non-cultured) per well in a 96-well V-bottom plate (Table 5).
    i. For the live/dead fixable blue single-stained control: expose 50,000 PBMCs to hot water (easiest to do this in a sink) for at least 60 seconds to induce cell death. Combine these PBMCs with 50,000 viable PBMCs.
  b. Vortex anti-mouse Ig and negative control compensation beads for at least 30 seconds and add 1 droplet of each per 96-well (Table 5).
  c. Stain the PBMCs/beads according to **steps 9f-o.**

**Table 3:** Preparation of life-dead staining mix

Reagent	Volume (μL/reagent)
Live/dead fixable blue (1:1000)	0.2
PBS	99.8
**Total**	**100**

**Table 4:** Preparation of membrane staining mix

Reagent	Volume (μL/reagent)
Alexa Fluor 700 anti-human CD45 antibody (1:800)	0.13
Brilliant Violet 711 mouse anti-human CD3 antibody (1:200)	0.50
Brilliant Violet 510 mouse anti-human CD8α antibody (1:200)	0.50
Phycoerythrin-Cyanine 7 mouse anti-human CD4 antibody (1:800)	0.13
Fluorescein Isothiocyanate mouse anti-human CD103 antibody (1:20)	5.00
Super Bright 780 mouse anti-human CD69 antibody (1:100)	1.00
Brilliant Violet 650 mouse anti-human CXCR6 antibody (1:100)	1.00
Allophycocyanin mouse anti-human CD62L antibody (1:100)	1.00
Brilliant stain buffer plus (1:10)	10.00
FACS buffer	80.74
**Total**	**100.00**

**Table 5:** Preparation of reference controls

Well	PBMCs/beads	Reagent for single-stained control	Volume (μL/reagent)
1	PBMCs	Unstained	N/A
2	PBMCs	Live/dead fixable blue	0.20
3	PBMCs	AF700 α-CD45 (1:800)	0.13
4	PBMCs	BV711 α-CD3 (1:200)	0.50
5	PBMCs	BV510 α-CD8α (1:200)	0.50
6	PBMCs	PE-Cy7 α-CD4 (1:800)	0.13
7	Beads	Unstained	N/A
8	Beads	FITC α-CD103 (1:20)	5.00
9	Beads	SB780 α-CD69 (1:100)	1.00
10	Beads	BV650 α-CXCR6 (1:100)	1.00
11	Beads	APC α-CD62L (1:100)	1.00

### Single-cell RNA sequencing of TRMs

**Timing: Variable depending on the number of experimental conditions and/or donors; maximum duration is 4 h, excluding cell partitioning and library preparation**

This section outlines a protocol for the fixation, transcriptomic analysis and assessment of TRMs of PBMCs at baseline (pre-culture) and PBMCs cultured for 21 days (regarding TRM cell numbers, day 14 can also be used) with or without the 3D NEM using the 10× Flex kit. This experiment was performed with cells derived from 1 donor.

12. Fixation of *in vitro* established 3D NEM and PBMC co-cultures
  a. For baseline samples: thaw cryopreserved PBMCs (**steps 4a-h**) derived from the same donors (autologous) as the 3D NEM and PBMCs used in the co-culture experiments.
  b. For day 21 TRMs: repeat **sections** 7 and 8; we used co-cultures which were 21 days old, but time-point day 14 can also be used. Transfer cells from PBMC + 3D NEM conditions through a 40 μm strainer to filter out the epithelial cells.
    ***Note:*** Sample preparation was performed according to manufacturer’s protocol using the Chromium Next GEM single cell fixed RNA sample preparation kit (My Document).
  c. Samples were resuspended in 1 mL PBS with 0.04% BSA and transferred to 1.5 mL microcentrifuge tubes.
  d. Centrifuge at 4°C, 400 x g for 5 min.
  e. Remove the supernatant without disturbing the pellet.
    ***Note:*** Up to 30 μL supernatant may be left behind.
  f. Add 500 μL Fixation buffer B (at 19°C) per pellet and resuspend by pipetting 5 times up and down.
  g. Incubate for 16–24 h at 4°C.
  h. Add 500 μL additive C (at 19°C) to the sample in Fixation Buffer B and resuspend by pipetting 5 times up and down.
  i. Centrifuge at 19°C, 850 x g for 5 min.
  j. Remove the supernatant without disturbing the pellet.
  k. Add 1 mL chilled Quenching Buffer B to the sample pellet, resuspend by pipetting 5 times up and down, and keep samples on ice.
  l. Determine cell concentration of the fixed sample and proceed immediately to the GEM-X Flex Gene Expression protocol.
    i. Store samples at 4°C for up to 1 week or at −80°C for up to 12 months (see manufacturer’s protocol for sample storage guidance).
13. Automated cell partitioning
  a. Use the Chromium GEM next gen chip and barcode 10,000 cells per sample following the manufacturer’s protocol.
  b. Use 3 lanes: 1) PBMCs cultured for 21 days, 2) PBMCs and 3D NEM cultured for 21 days and 3) baseline PBMCs.
14. Library generation
  a. Library generation was performed with the Chromium Fixed RNA kit Human Transcriptome, 4rxns, according manufacturer’s instructions.
  b. Sequencing was performed on a partial lane of the NovaSeq 6000 PE150bp. The expected sequence depth for gene expression is 20,000 reads/cell.
15. Our recommendations for the data analysis
  a. The 10× cellranger output was analyzed and clustered using Seurat package v5 in R.^9^
  b. Clusters were manually annotated using marker genes. To identify TRM clusters, the enrichment of the TRM core gene signature was tested for each cluster using the FGSEA package, as described.^10^

### Functional assessment of TRMs

**Timing: 24 h**

This section provides a step-by-step protocol of the functional assessment of T cells within the 3D NEM and immune cell co-culture. Phorbol 12-myristate 13-acetate (PMA) and ionomycin are known to induce (non-specific) signaling and subsequent potent cytokine production. These were used in our 3D NEM and PBMC co-cultures to determine cytokine production of TRM-like cells and their non-TRM counterparts.

16. Stimulating *in vitro* established 3D NEM and PBMC co-cultures with PMA and ionomycin
  a. Repeat **sections** 7 and 8; we used co-cultures which were 21-days old, but time-point day 14 can also be used.
  b. Transfer the cells through a 40 μm strainer in a new conical (V)-bottom plate to filter out the epithelial cells.
  c. Count the cells.
    i. Mix 20 μL cell suspension with 20 μL trypan blue and transfer 10 μL into a Bürker Türk counting chamber.
  d. Make triplicates per sample and plate 50,000 cells 100 μL T cell medium per 96-well in a round-bottom plate.
  e. Add 100 μL T cell medium with 100 ng/mL PMA and 1 mg/mL ionomycin (final concentration in well) per 96-well.
  f. Incubate for 1 hour in an incubator (37°C, 5% CO_2_).
  g. Add 5 μL T cell medium with 10 ng/mL Brefeldin A (final concentration in well).
  h. Incubate for 4 hours in an incubator (37°C, 5% CO_2_).
17. Staining and measuring cytokine-producing TRMs
  a. Collect the plate(s) from the incubator and transfer cells to a 96-well V-bottom plate.
  b. Stain the cells with a viability dye as described in Table 3 and with extracellular monoclonal antibodies described in Table 6 (follow **steps 9a-m** and prepare controls as explained in steps **11a-c**).
  c. Permeabilize the cells with the Foxp3/Transcription Factor Staining Buffer Set, according to the manufacturer’s instructions.
    i. Resuspend cells per condition in 180 μL of FoxP3 fixation/permeabilization working solution.
    ii. Incubate for 30 min at 19°C.
    iii. Centrifuge at 4°C, 800 × *g* for 5 min and discard the supernatant.
    iv. Resuspend cells per condition in 150 μL of 1× perm/wash.
    v. Centrifuge at 4°C, 800 × *g* for 5 min and discard the supernatant by carefully pipetting off the remaining liquid. Wash 2 more times.
  d. Remove supernatant and add 100 μL of intracellular staining mix (Table 7) per sample.
  e. Cover the plate with aluminum foil and incubate on ice for 15 min.
  f. Add 50 μL of 1× perm/wash to each sample and centrifuge at 4°C, 800 × *g* for 5 min.
  g. Add 150 μL of 1× perm/wash to each sample and centrifuge at 4°C, 800 × *g* for 5 min.
  h. Resuspend cells with 200 μL FACS buffer per sample and transfer to 1.4 mL U-bottom tubes.
  i. Add 100 μL FACS buffer per tube (total volume is 300 μL).
  j. Measure the cells with a Cytek Aurora.

**Table 6:** Preparation of membrane staining mix

Reagent	Volume (μL/reagent)
Allophycocyanine mouse anti-human CD3 antibody (1:20)	5.00
Phycoerythrin mouse anti-human CD8α antibody (1:100)	1.00
Fluorescein Isothiocyanate mouse anti-human CD103 antibody (1:20)	5.00
Phycoerythrin-Cyanine 7 mouse anti-human CD69 antibody (1:20)	5.00
Brilliant stain buffer plus (1:10)	10.00
FACS buffer	74.00
**Total**	**100.00**

**Table 7:** Preparation of intracellular staining mix

Reagent	Volume (μL/reagent)
BV605 α-IFNγ (1:200)	0.50
PerCP-Cy5.5 α-TNFα (1:100)	1.00
FACS buffer	98.50
**Total**	**100.00**

## Expected outcomes

Following culture initiation, 3D NEM formation typically becomes evident within the first week. We have decided to use the term ‘3D NEM’ throughout the protocol, as the terminology ‘spheroid’ and ‘organoid’ is not clear. The protocol we developed creates 3D NEM with an apical-in phenotype. After the first passage, the frequency of passaging can be increased based on growth kinetics, but this is highly variable per donor. We recommend plating epithelial cells at a high density (Figures 2D and 2E) rather than seeding them too sparsely (Figures 2A and 2B). To preserve the culture and mitigate potential loss, it is strongly recommended to cryopreserve backup vials of a low passage number once the 3D NEM is established. We have used epithelial cell cultures not older than passage 9 and we recommend to use cultures that were passaged at least once.

The 3D NEM typically start to differentiate 14–28 days following culture initiation or passaging. The differentiation status can be assessed by confocal microscopy using fluorescently labeled antibodies specific for goblet cells (mucin 5AC), ciliated cells (alpha tubulin), and club cells (CC10). Differentiation of the 3D NEM is characterized by the presence of all three cell types and the formation of a central lumen lined by inward-facing ciliated cells and visible cilia (Figures 3B–3D). Increased differentiation, and therefore ciliation, is observed in 3D epithelial cell cultures over time (when not passaged, Figure 3), with reduced capacity to expand.^11^

**Figure 5:**
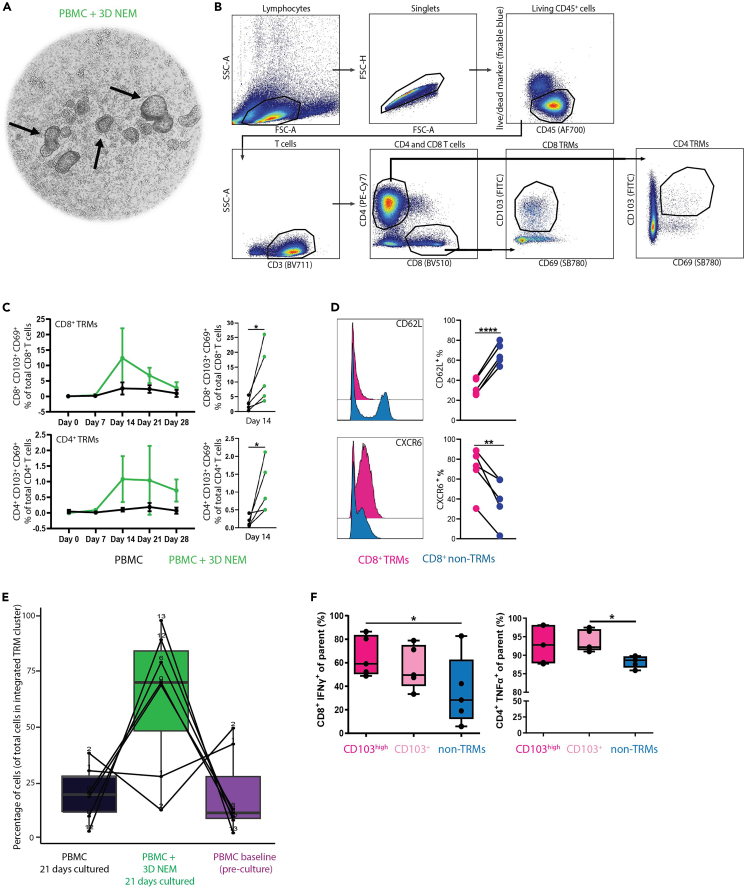
Analysis of TRMs derived from the *in vitro* 3D NEM and autologous immune cell co-culture (A–F) The human-derived 3D NEM was co-cultured with PBMCs (20.000 epithelial cells and 100,000 PBMCs per condition) for 28 days. Cells were cultured in T cell medium, which was refreshed every 2-3 days. TRM phenotype was analyzed weekly using spectral flowcytometry or at day 21 using scRNAseq and their cytokine profile was determined using an intracellular flow cytometry cytokine staining after 16h PMA and ionomycin stimulation. (A) Brightfield image of a representative PBMC + 3D NEM co-culture on day 7 (5× magnification). Black arrows highlight three examples of 3D nasal epithelial structures. (B) Gating strategy of TRMs within CD8^+^ and CD4^+^ T cell populations. (C) Frequencies of CD8^+^ and CD4^+^ TRMs in PBMC or PBMC + 3D NEM shown over time (left panel, data represents mean ± SEM) or on day 14 (right panel) were tested using multiple individual donors in three independent experiments (day 7: n=2, day 14: n=5, day 21: n=5 and day 28: n=4). Conditions were plated in triplicate and means per condition per donor was calculated. (D) Frequency of CD62L^+^ and CXCR6^+^ CD8^+^ TRMs measured on day 14 shown as histogram (n=1 representative donor) and quantitative graphs (n=5 individual donors). (E) To study enrichments of TRM genes, single cell RNA sequencing was performed on cells cultured in 2 conditions versus thawed PBMCs. Seurat clustering was performed on the integrated dataset of these conditions filtered on CD4^+^ T cells. The relative frequency of cells within each TRM cluster (n=7 TRM clusters out of total 18 clusters) is shown across the different conditions. (F) Shown are frequencies (%) of CD8^+^ IFNγ^+^ T cells (left panel) and CD4^+^ TNFα^+^ T cells (right panel) within the with PMA and ionomycin stimulated CD103^high^, CD103^+^ or CD103^-^ populations. ∗ represents P = ≤ 0.05, ∗∗ represents P = ≤ 0.01 and ∗∗∗∗ represents P = ≤ 0.0001, calculated using a paired *t* test.

Co-culturing differentiated 3D NEM with unstimulated autologous PBMCs (Figure 5A) changes the phenotype of these blood-derived T cells, as they start expressing markers related to TRMs. Nasal-derived CD8 TRM subsets are known to mainly co-express CD69 and CD103, whereas CD4 TRM subsets are known to express only CD69 or co-express CD69 and CD103.^12^ These T cell subsets are found *in vivo* within the nasal mucosa where they reside and play an important role in the defense against recurrent pathogens.^13^ We used the gating strategy shown in Figure 5B to identify CD8^+^ and CD4^+^ TRMs. Lymphocytes are identified based on size (forward scatter, FSC-A) and granularity (sideward scatter, SSC-A), which is followed by exclusion of doublets, dead and CD45 negative cells. CD8^+^ and CD4^+^ T cells were selected from CD3-expressing cells, and TRMs were gated based on CD69^+^ and CD103^+^ double positive events. Figure 5C shows the frequency of TRM-like cells in the co-culture model over time. TRMs are not detected in the circulation, and not expected to be present in PBMC samples at the baseline time-point. Over time, an increase of TRM-like cells is observed in PBMC + 3D NEM co-culture conditions, which peaks around day 14–21. This is not observed in the PBMC control conditions. It is shown in literature that CD8^+^ TRMs express low levels of CD62L,^14^ and express CXCR6.^15^ We find similar expression patterns of these markers on CD8^+^ TRM-like cells in our co-culture system compared to their non-TRM counterparts (Figure 5D), which confirms that these cells are phenotypically similar to reported literature. Furthermore, scRNAseq combined with GSEA revealed that the majority of T cell clusters found in the 21 day old PBMC + 3D NEM co-culture conditions were significantly enriched for TRM genes compared to PBMCs alone (21 days cultured and baseline; Figure 5E). A hallmark for TRMs is the capacity to produce high levels of IFNγ and TNFα.^16^ PMA and ionomycin-stimulated day 21-derived TRM-like T cells (identified by CD103 expression, due to the upregulation of CD69 upon stimulation) produced significantly more IFNγ (CD8^+^ T cells) and TNFα (CD4^+^ T cells) compared to CD103^-^ (non-TRM) cells (Figure 5F). This finding indicates that the *in vitro* generated TRM-like cells are functionally more active compared to non-TRM cells by an enhanced cytokine production.

## Limitations

The variable yield of nasal epithelial cells obtained via nasal scraping impacts the efficiency of the generation of a 3D NEM. For some donors, generating a 3D NEM may not be achievable if epithelial cell counts after harvest are too low (see troubleshooting problem 2). Additionally, donor-specific differences in epithelial cell proliferation can impact model formation as well as the time required to establish them. In certain cases, low proliferation rates result in failed cell models. These limitations directly impact the number of epithelial cells available for the downstream TRM induction experiments. Moreover, this assay requires the use of autologous PBMC and nasal epithelial cells, meaning that nasal cells cannot be obtained from commercial sources due to HLA-mismatching.

Considerable variability in TRM induction has been observed across technical replicates. To reduce experimental variability, we recommend to use the average of three technical replicates per condition per donor. The induction of CD4^+^ TRM-like cells without expression of CD103 remains difficult to investigate using flow cytometry, due to upregulated expression of CD69 for cultured PBMCs in absence of autologous epithelial cells.

Moreover, long-term co-culture of more than 28 days was not possible, as 3D nasal epithelial cell structures start losing structural integrity and then the frequency of TRMs starts decreasing after 21 days.

## Troubleshooting

### Problem 1

The three BME-2 droplets are merged together and/or against the side of the 24-well (Figure 1). This will impair diffusion of growth factors into the BME-2 and thereby potentially compromising the growth of the 3D NEM.

### Potential solutions

Do not retrieve and re-pipet the BME-2 droplets once they are in the plate, as this will result in a significant loss of cells and may induce cellular stress or apoptosis. Instead, implement the following solutions the next time you passage the epithelial cell culture. It is critical to pre-warm the 24-well plate in a 37°C incubator. Pipetting BME-2 droplets into a warm 24-well plate facilitates rapid solidifying of the droplets, thereby minimizing spreading. For optimal results, keep the 24-well plate flat and your pipet straight and try to find the best locations in the 24-well to prevent contact with adjacent droplet or the side of the well. Repeated practice will improve precision. It is also recommended to be careful handling the plate and shutting the door of the incubator, as fast movements can disrupt BME-2 droplets. Alternatively, pipette two droplets per 24-well instead of three. This will however impact the costs, as you would need more wells, but it makes it easier to keep the droplet from merging into each other or the wall of the well.

### Problem 2

Low levels of epithelial cell numbers after harvest.

### Potential solution

Resuspend cells in 30 μL BME-2, rather than 90 μL, and pipette 1 droplet to enhance cell concentration. Additionally, the collection of nasal epithelial cells using FLOQSwabs (Copan, 220252) or nasal brushing will significantly increase the cell yield compared to nasal curette sampling.

### Problem 3

Part of the BME-2 droplet detached during culture. This could potentially lead to cell loss when replacing airway epithelial medium.

### Potential solutions

Be extremely cautious when replacing airway epithelial medium; carefully aspirate to avoid disrupting or aspirating portions of the BME-2 droplet, and fresh medium must be added gently. If the BME-2 droplets are no longer intact, the culture should be re-plated. Otherwise, continue the culture until its ready for passaging. Implement the following solutions to avoid the problem. Potential medium residues can dilute BME-2, therefore it is critical to completely remove medium before resuspending the epithelial cell pellet in BME-2 (**step 1r**). Minimize the time culture plates spend outside the incubator, as prolonged exposure to lower temperatures may soften the BME-2 droplets, impairing their structural stability.

## Resource availability

### Lead contact

Further information and requests for resources and reagents should be directed to and will be fulfilled by the lead contact, Lisa A. King (l.a.king@lumc.nl).

### Technical contact

Technical questions on executing this protocol should be directed to and will be answered by the technical contact, Lisa A. King (l.a.king@lumc.nl).

### Materials availability

This study did not generate new unique reagents.

### Data and code availability

• All data reported in this paper can be shared by the lead contact upon reasonable request.
• This paper does not report original code.
• Any additional information required to reanalyze the data reported in this paper is available from the lead contact upon request.

## Acknowledgments

This project has received funding from the 10.13039/501100010767Innovative Medicines Initiative 2 Joint Undertaking under grant agreement no. 101007799 (Inno4Vac). This Joint Undertaking receives support from the European Union’s Horizon 2020 research and innovation program and EFPIA. This work was further supported by an NWO grant (OCENW.KLEIN.461) (S.P.J.) and by the Lung Foundation Netherlands: AWWA grant #12.0.17.001 (J.S.S.).

We would like to thank Alicia de Kroon and Sanne Steenbergen for their assistance in the processing of PBMCs and nasal epithelial samples obtained from participants in the TINO study and Ianthe Rebergen for her assistance with the revision experiment. We also thank the research nurses of the Clinical Research Unit for Internal Medicine (KRIG) at the Leiden University Medical Center (LUMC, Netherlands) for sampling the TINO participants. The authors also acknowledge the Flow Cytometry Core Facility at LUMC (Leiden, the Netherlands; Flow Cytometry Core Facility | LUMC) for technical support in the flow cytometry studies.

The graphical abstract is created in BioRender. King, L. (2026) https://BioRender.com/tuf85qt.

## Author contributions

S.P.J. obtained funding. P.S.H., A.M.v.d.D., S.P.J., and W.H. supervised the project. L.A.K., I.v.d.V., J.S.S., and W.H. performed practical work. L.A.K. and Y.S. analyzed the data. L.A.K. drafted and wrote the manuscript. I.v.d.V., J.S.S., G.H.G., P.S.H., A.M.v.d.D., S.P.J., and W.H. reviewed the manuscript.

## Declaration of interests

The authors declare no competing interests.
